# Risk factors for peritonitis in patients on continuous ambulatory peritoneal dialysis who undergo colonoscopy: a retrospective multicentre study

**DOI:** 10.1186/s12876-019-1081-2

**Published:** 2019-11-06

**Authors:** Tae-Geun Gweon, Sung Hoon Jung, Sang Woo Kim, Kang-Moon Lee, Dae Young Cheung, Bo-In Lee, Hwang Choi

**Affiliations:** 1 Division of Gastroenterology, Department of Internal Medicine, College of Medicine, The Catholic University of Korea, Seoul, Republic of Korea; 2 Department of Internal Medicine, College of Medicine, Eunpyeong St. Mary’s Hospital, The Catholic University of Korea, Seoul, Republic of Korea

**Keywords:** Peritoneal dialysis, Continuous ambulatory peritoneal dialysis, Colonoscopy, Peritonitis

## Abstract

**Background:**

Colonoscopy is associated with a risk of peritonitis in patients on peritoneal dialysis. However, no study has yet described the risk factors in play.

**Methods:**

This was a retrospective multicentre study. The medical records of patients on continuous ambulatory peritoneal dialysis (CAPD) who underwent colonoscopy from January 2003 to December 2012 were analysed. We recorded demographic characteristics, colonoscopic factors, use of prophylactic antibiotics, and development of peritonitis. Colonoscopy-related peritonitis was defined as peritonitis developing within 1 week after colonoscopy. Demographic and clinical characteristics were compared between patients who did and those who did not develop peritonitis.

**Results:**

During the study period, 236 patients on CAPD underwent colonoscopy, of whom 9 (3.8%) developed peritonitis. The rates of polypectomy/endoscopic mucosal resection were significantly higher in the peritonitis group than in the no peritonitis group (66.7 vs. 23.4%, *p =* 0.009). Prophylactic antibiotics were prescribed before colonoscopy in 65 patients; none developed peritonitis. No patient who developed peritonitis received prophylactic antibiotics (*p =* 0.067).

**Conclusions:**

Advanced procedures including polypectomy or endoscopic mucosal resection increase colonoscopy-related peritonitis in patients on CAPD. Randomized controlled trials to investigate whether prophylactic antibiotics are needed to prevent peritonitis in all CAPD patients are warranted.

## Introduction

Peritoneal dialysis (PD) is a major form of renal replacement. Peritonitis is an important complication associated with technical failure and death, as well as an important quality measure, in patients on PD. [1–4] The International Society for Peritoneal Dialysis recommends an annual peritonitis rate of less than 0.5 episodes per year [5]. Risk factors for PD-related peritonitis are older age, diabetes, hypoalbuminemia, and invasive procedures including hysteroscopy, dental procedures, and colonoscopy [5–9].

As most gut microorganisms are found in the colon, the gut is a potential source of intra-abdominal infection [10]. Several studies have reported the development of peritonitis after colonoscopy in patients on PD. [11, 12] Recently, the American Society of Gastrointestinal Endoscopy and the International Society for Peritoneal Dialysis recommended that prophylactic antibiotics be prescribed before colonoscopy for such patients [5, 13]. However, little evidence supports this recommendation. One study found that the rate of peritonitis was 6.4% in PD patients undergoing endoscopy [14]. However, the cited work included patients undergoing upper endoscopy and hysteroscopy as well as colonoscopy [14]. Moreover, previous studies could not identify factors contributing to colonoscopy-related peritonitis because of a small sample size [12, 14, 15]. In this multicentre study, we sought to identify factors associated with peritonitis and the effects of antibiotic prophylaxis in patients on PD undergoing colonoscopy.

## Materials and methods

### Study population and methods

This was a multicentre, retrospective, cohort study. The medical records of patients on continuous ambulatory peritoneal dialysis (CAPD) who underwent colonoscopy from January 2003 to December 2012 were analysed. Patients were treated in seven hospitals of the Catholic University of Korea: Incheon St Mary’s Hospital, Vincent Hospital, Bucheon St. Mary’s Hospital, Yeouido St. Mary’s Hospital, Uijeongbu St. Mary’s Hospital, and Seoul St. Mary’s Hospital. The study protocol was approved by the institutional review board of each participating hospital. Written informed consent was waived because the work was retrospective in nature. We recorded demographic characteristics and colonoscopy-related factors, including the indication for colonoscopy, bowel preparation quality, biopsy status, and the need for advanced procedures including polypectomy or endoscopic mucosal resection (EMR) during colonoscopy. The use of prophylactic antibiotics was assessed. All patients ingested 4 L of PEG (Colyte, Taejoon Pharma, Seuol, Korea). Patients were divided into the peritonitis and no peritonitis groups. In those who developed colonoscopy-related peritonitis, the results of peritoneal fluid culture and antibiotic treatment, and the clinical outcomes, were investigated.

### Definitions

Colonoscopy-related peritonitis was defined as peritonitis developing within 1 week after colonoscopy. Peritonitis was diagnosed when at least two of the following criteria were met: (1) abdominal pain with or without a cloudy dialysis effluent, (2) a peritoneal effluent white cell count > 100/μL with > 50% polymorphonuclear neutrophils, and (3) a positive dialysis effluent culture [5].

### Statistical analysis

Continuous variables are presented as means ± standard deviations and were compared using Student’s *t*-test or the Mann–Whitney U-test. Categorical variables are presented as numbers with percentages and were compared using the chi-squared or Fisher’s exact test. Demographic and clinical characteristics were compared between patients who did and those who did not develop peritonitis. A *p*-value < 0.05 was considered significant. All statistical analyses were performed using SAS ver. 9.0 software (SAS Institute, Cary, NC).

## Results

### Baseline characteristics of the study subjects

During the study period, 236 patients on CAPD underwent colonoscopy after removing dialysate, of whom 9 (3.8%) developed peritonitis. The patient baseline characteristics are listed in Table 1. Sex, age, and body mass index were comparable between the two groups. The diabetes rates were 33.3% in the peritonitis group and 37.4% in the no peritonitis group (*p* = 1.000). The CAPD durations were 33.9 and 51.4 months, respectively. The screening colonoscopy rates were 55.6% in the peritonitis group and 60.4% in the no peritonitis group (*p* = 0.744).

**Table 1:** Baseline characteristics

Characteristics	Peritonitis (*n* = 9)	No peritonitis (*n* = 227)	*P*
Male, n (%)	5 (55.6)	123 (54.2)	1.000
Age, years (± SD)	52.6 (± 11.1)	50.4 (± 16.7)	0.061
BMI, kg/m^2^ (± SD)	24.3 (± 2.2)	24.2 (± 3.3)	0.796
Etiology of ESRD			1.000
Diabetes, n (%)	3 (33.3)	85 (37.4)	
Non-diabetes, n (%)	6 (66.7)	142 (62.6)	
Duration of CAPD, months	33.9 ± 25.5	51.4 ± 53.7	0.207
Indication for colonoscopy			
Screening, n (%)	5 (55.6)	137 (60.4)	0.744
Non-screening, n (%)	4 (44.4)	90 (39.6)	

*SD* standard deviation, *ESRD* end stage renal disease, *CAPD* continuous ambulatory peritoneal dialysis

### Colonoscopic factors and the use of prophylactic antibiotics

The results of colonoscopy are shown in Table 2. Neither the experience of expert or trainee nor bowel preparation quality differed between the two groups. The colonic mucosa was manipulated/biopsied, and advanced procedures such as polypectomy or EMR performed, in 123 patients. The extent of colonic mucosal manipulation was higher in the peritonitis group than in the no peritonitis group (88.9 vs. 50.7%, *p* = 0.037). Colonic mucosal biopsy did not increase the rate of peritonitis development (peritonitis vs. no peritonitis group: 22.2% vs. 27.3%, *p =* 1.000). There was no association between the size of polyps and the infection rate in polypectomy/EMR (peritonitis vs. no peritonitis group: 0.97 cm vs 0.96 cm, *p =* 0.962). However, the rates of polypectomy/EMR were significantly higher in the peritonitis group than in the no peritonitis group (66.7 vs. 23.4, *p =* 0.009). Prophylactic antibiotics were prescribed before colonoscopy to 65 patients (27.5%), and none of these patients developed peritonitis. However, the proportion of patients who received prophylactic antibiotics prior to colonoscopy did not differ significantly between the two groups (peritonitis vs. no peritonitis group: 0 vs. 28.6%; *p =* 0.067). In a subgroup analysis, of the 59 patients who underwent polypectomy or EMR, prophylactic antibiotics were given to 14 (23.7%). Although none of the six patients who developed peritonitis after polypectomy or EMR received prophylactic antibiotics, such antibiotics did not prevent peritonitis statistically (peritonitis vs. no peritonitis group: 0 [0/6 vs. group 26.4% [14/53], *p =* 0.319).

**Table 2:** Factors related to colonoscopy

Characteristics	Peritonitis (n = 9)	No peritonitis (n = 227)	*P*
Colonoscopist, n (%)			0.505
Expert, n (%)	3 (33.3)	118 (52.0)	
Trainee, n (%)	6 (66.7)	109 (48.0)	
Bowel preparation quality n (%)			
Excellent or good	9 (100)	216 (95.2)	
Fair	0 (0)	11 (4.8)	
Colonoscopic procedure	8 (88.9)	115 (50.7)	0.037
Colon biopsy, n (%)	2 (22.2)	62 (27.3)	1.000
Polypectomy / EMR, n (%)	6 (66.7)	53 (23.4)	0.009
Use of prophylactic anbitiobitcs, n (%)			0.067
Yes	0 (0)	65 (28.6)	
No	9 (100)	162 (71.4)	

*EMR* endoscopic mucosal resection

### Clinical outcomes

The details of the peritonitis cases are listed in Table 3. The microorganisms isolated from peritoneal effluent were *Staphylococcus aureus* (*n* = 1), *Escherichia coli* (*n* = 5), and none (*n* = 3). One patient who underwent no advanced procedure had *S. aureus*. All patients received at least two antibiotics. The PD catheter was removed from one patient 5 days after antibiotic treatment commenced. We recorded no mortalities.

**Table 3:** Details of peritonitis

Patient number	Sex	Age	Procedure	Culture	Treatment	Outcome
1	Female	20–30	Colon biopsy	No growth	Cefazolin + gentamycin	Recovered
2	Male	30–40	EMR	No growth	Cefazolin + gentamycin	Recovered
3	Male	30–40	Polypectomy	*E. coli*	Vancomycin + ceftazidime	Recovered
4	Male	40–50	EMR	*E. coli*	Cefamezine + tobramycin	Recovered
5	Female	50–60	EMR	No growth	Cefamezin + gentamycin	Recovered
6	Male	50–60	EMR	*E. coli*	Cefamezin + gentamycin	Recovered
7	Male	60–70	Polypectomy	*E. coli*	Cefazolin + gentamycin	Recovered
8	Female	60–70	Colon biopsy	*E. coli*	Ceftazidime + metronidazole	Recovered
9	Female	70–80	No procedure	*S. aureus*	Cefazolin + gentamycin	Catheter removal

*EMR* endoscopic mucosal resection

## Discussion

We sought to identify risk factors for colonoscopy-associated peritonitis in patients on CAPD. The overall peritonitis rate was 3.8%. Both polypectomy and EMR were peritonitis risk factors. Although statistical significance was not attained (*p* = 0.067), peritonitis was absent in patients who received antibiotic prophylaxis prior to colonoscopy. To the best of our knowledge, this is the first multicentre study to explore the risk factors for colonoscopy-related peritonitis in patients on CAPD; we included the largest number of patients evaluated on this subject to date.

The principal causes of PD-related peritonitis are catheter infections, thus contamination of PD catheters and exit site and tunnel infections. Less often, microorganisms from the colon or vagina, or haematogenous dissemination after dental procedures, trigger peritonitis in patients on PD. [16, 17] We found that polypectomy and EMR were risk factors for peritonitis. The colonic mucosa prevents microorganism translocation and controls intestinal permeability [18, 19]. Polypectomy and EMR create colonic mucosal defects facilitating translocation of intestinal microorganisms. We found that colonic biopsy was not associated with a risk of peritonitis. Such mucosal defects may be smaller than those caused by polypectomy or EMR. Also, we removed colon polyps electrically, thus not via cold snaring. Compared with cold-snare polypectomy, hot-snare polypectomy and EMR damage the large bowel wall to greater extents [20, 21]. Thermal injury of the colonic mucosa may act synergistically with a mucosal defect to trigger peritonitis.

Of the 113 patients who did not undergo mucosal manipulation, peritonitis occurred in only 1 (0.8%). The causative organism was *S. aureus*. The causative organism depends on the infection site. Usually, Gram-positive bacteria cause catheter-related infections. However, Gram-negative bacteria are commonly translocated from the colon or vagina [5]. *S. aureus* is the most common causative agent of catheter-related peritonitis [16]; we could not exclude the possibility of catheter-related peritonitis in the abovementioned patient. Peritonitis resolved after catheter removal.

Were prophylactic antibiotics useful? Of the 236 patients, only 65 received such antibiotics. The overall peritonitis rate after colonoscopy was 3.8%. When we divided the patients into those who received prophylactic antibiotics and those who did not, the peritonitis rates were 0 (0/65) and 5.3% (9/171), respectively. Although statistical significance was not attained (*p* = 0.067), peritonitis did not develop in any patient who received prophylactic antibiotics, in line with the findings of previous studies [12, 14]. Most studies found that the use of prophylactic antibiotics did not attain statistical significance in terms of peritonitis development. It is unethical to give patients placebos. We included patients on CAPD who underwent colonoscopy from 2003 to 2012, of whom a relatively small proportion (27.5%) received antibiotics prior to colonoscopy. We gave antibiotics prior to colonoscopy from 2010. Colonic neoplasms are found in up to 50% of patients undergoing colonoscopy [22–25]. Although diagnostic colonoscopy lacking a therapeutic procedure may not cause peritonitis, physicians cannot predict the presence of colon polyps. Therefore, prophylactic antibiotics should be given to all patients on CAPD prior to colonoscopy.

Our study had several strengths. First, this is the first multicentre study to explore whether colonoscopy triggers peritonitis in patients on PD. Second, we investigated factors causing peritonitis and identified advanced procedures such as polypectomy and EMR as triggers.

Several limitations of the study should be addressed. The work was retrospective in nature. Some data were lacking. Colonoscopy procedure time, which might affect peritonitis development, was not recorded. We did not include patients on automated PD (APD), but rather only CAPD patients. Peritoneal fluid triggers peritonitis. As patients on APD do not retain peritoneal fluid during the day, we hypothesized that patients on CAPD are at a higher risk of colonoscopy-related peritonitis than are patients on APD; thus, our findings may not be applicable to patients on APD. In addition, we just surveyed the use of prophylactic antibiotics, not antibiotic regimens. Further studies of prophylactic antibiotic regimens are needed to prevent colonoscopy-related peritonitis in CAPD patients.

## Conclusions

Advanced procedures including polypectomy and EMR increase the risk of colonoscopy-associated peritonitis in patients on CAPD. Randomized controlled trials to investigate whether prophylactic antibiotics are needed to prevent peritonitis in all CAPD patients are warranted.

## Data Availability

The datasets generated or analysed during the current study are available from the corresponding author on reasonable request.

## Abbreviations

CAPD: Continuous ambulatory peritoneal dialysis
EMR: Endoscopic mucosal resection
PD: Peritoneal dialysis
